# Diagnostic accuracy of Xpert MTB/RIF Ultra and culture assays to detect *Mycobacterium Tuberculosis* using OMNIgene-sputum processed stool among adult TB presumptive patients in Uganda

**DOI:** 10.1371/journal.pone.0284041

**Published:** 2023-04-21

**Authors:** Abdulwahab Sessolo, Emmanuel Musisi, Sylvia Kaswabuli, Josephine Zawedde, Patrick Byanyima, Wilber Sabiiti, Stanley Walimbwa, Joseph Ola, Ingvar Sanyu, Rejani Lalitha, Moses Kamya, Lucian Davis, William Worodria, Laurence Huang

**Affiliations:** 1 Infectious Diseases Research Collaboration, Kampala, Uganda; 2 Department of Biochemistry and Sports Science, Makerere University, Kampala, Uganda; 3 Division of Infection and Global Health, School of Medicine, University of St. Andrews, Scotland, United Kingdom; 4 China Uganda Friendship Hospital Naguru, Kampala, Uganda; 5 Department of Internal Medicine, Makerere University College of Health Sciences, Kampala, Uganda; 6 Department of Epidemiology of Microbial Diseases, Yale School of Public Health, New Haven, CT, United States of America; 7 Pulmonary, Critical Care and Sleep Medicine, Yale School of Medicine, New Haven, CT, United States of America; 8 Division of Pulmonary and Critical Care Medicine, University of California San Francisco, San Francisco, CA, United States of America; 9 Division of HIV, Infectious Diseases and Global Medicine, University of California San Francisco, San Francisco, CA, United States of America; Hangzhou Red Cross Hospital, CHINA

## Abstract

**Background:**

Stool is a potential sample for diagnosing *Mycobacterium tuberculosis* (*Mtb*) in patients with difficulty in expectorating. However, high mycobacterial culture contamination rates and Xpert MTB/RIF Ultra test error rates on stool samples have limited its use. OMNIgene SPUTUM (OM-S) is a sample transport reagent with characteristics of sputum decontamination while maintaining viable *Mtb*. We evaluated the impact of OM-S on *Mtb* diagnostic yield from stool using smear microscopy, Xpert MTB/RIF Ultra, and culture among presumptive TB patients.

**Methods:**

Paired stool and expectorated sputum samples were collected from consecutive Ugandan adults undergoing diagnostic evaluation for pulmonary TB between June 2018 and June 2019. Stool was divided into 2 portions: one was homogenized in OM-S (OM-S stool) and the other in PBS (PBS stool) as control. Both sputum and processed stool were tested for *Mtb* using concentrated smear fluorescence microscopy (CFM), Xpert MTB/RIF Ultra (Xpert) and Mycobacteria Growth Indicator Tube (MGIT) culture. Sensitivity, specificity, and predictive values for each test were calculated against sputum MGIT culture as the reference standard.

**Results:**

Of the 200 participants, 120 (60%) were male, 73 (37%) were HIV positive (median CD4 120 cells/uL (IQR 43–297)) and 128 (64%) had confirmed pulmonary TB by sputum MGIT culture. Seven (4%) OM-S stool Xpert samples reported errors while 47 (25%) and 103 (61%) were contaminated on OM-S stool MGIT and PBS stool MGIT, respectively. OM-S stool MGIT was able to accurately diagnose 56 of the contaminated PBS stool MGIT samples compared to only 5 of the contaminated OM-S stool MGIT samples diagnosed by PBS stool MGIT.

Sensitivity (95% Confidence Interval, CI) 89% (83–94) for OM-S stool Xpert was higher compared to that of OM-S stool MGIT 60% (51–69) and PBS stool MGIT 42% (32–52). Specificity (95%CI) 91% (82–97) was also higher for OM-S stool Xpert compared to OM-S stool MGIT 64% (51–75) and PBS stool MGIT 26% (16–38).

**Conclusion:**

Stool processed with OM-S showed potential to improve *Mtb* diagnostic yield and reduce rates of indeterminate results when tested on Xpert and MGIT culture. The method may thus be of value in *Mtb* detection among patients with difficulty to expectorate.

## Introduction

Tuberculosis (TB) is the leading cause of death from a single infectious agent worldwide, responsible for 1.4 million deaths annually [1]. During 2020, 90,000 individuals were estimated to have fallen ill with TB in Uganda but only 60,887 were notified possibly explained by a combination of interruption of TB care services by COVID pandemic lockdowns and decreased availability of diagnostic services. In 2018, TB caused death among 16,000 Ugandans necessitating an urgent intervention to reduce this burden [2]. Early and effective methods of diagnosis and treatment are critical to reduce TB morbidity and mortality. Expectorated sputum remains the standard sample for diagnosis of pulmonary TB (PTB), but some patients, especially young children, elderly adults, and severely ill HIV+ persons may not be able to expectorate sputum sufficiently, making diagnosis of PTB difficult in these sub-populations [3]. Alternative samples such as induced sputum, gastric aspirates, and nasopharyngeal aspirates have been used, but these samples are challenging to obtain because the collection procedures are invasive, operator-dependent, time-consuming, and relatively costly [4–7]. Thus, there is a substantial interest in the use of other, more easily accessible sample types.

Stool is one such specimen. Patients commonly swallow lower respiratory tract secretions after coughing. Some *Mycobacterium tuberculosis* (*Mtb*) bacilli or DNA may pass intact through the gastrointestinal tract into their stool. Prior studies have identified both live *Mtb* and dead *Mtb* DNA in stool using standard TB tests [8–16]. Stool is therefore a potential alternative or additional sample for TB diagnosis, however its use has been limited by bacterial overgrowth leading to high rates of contamination in mycobacterial culture, and inhibition of amplification in molecular tests leading to increased error rates [14]. In 2017, we evaluated the performance of OMNIgene SPUTUM (OM-S), a sample transport reagent (DNA Genotek, Inc, Ottawa, Canada) designed to preserve *Mtb* viability in sputum samples while allowing for storage and transport at ambient temperature without a need for cold chain. We found that OM-S treated sputum samples had three-fold lower sputum culture contamination rates on both solid and liquid media than the untreated samples. There was also a high concordance of results between OM-S treated and untreated samples on microscopy, Xpert, and culture tests [17].

Based on these findings, we developed a stool processing method incorporating OM-S and carried out a prospective cross-sectional study to evaluate its performance on concentrated smear fluorescence microscopy (CFM), Xpert MTB/RIF Ultra (Xpert) and Mycobacteria Growth Indicator Tube (MGIT) culture in adults undergoing diagnostic evaluation for active TB relative to a sputum-based reference standard. We hypothesized that (i) the OM-S based stool processing method would be associated with high diagnostic accuracy for *Mtb*, and (ii) reduced Xpert MTB/RIF Ultra error rates and culture contamination rates on stool.

## Methods and materials

### Study design, site, and participants

We conducted a prospective, cross-sectional study of consecutively presenting patients with possible TB signs and symptoms at China-Uganda Friendship Hospital Naguru in Kampala, Uganda between June 2018, and June 2019. This study was nested within the ongoing Inflammation, Aging, Microbes and Obstructive Lung Disease (I AM OLD) study, a longitudinal cohort study of adults with pneumonia followed for the development and progression of chronic obstructive pulmonary disease (COPD). Eligible participants were adults (≥18 years) presenting with cough with or without weight loss, chest pain, and/or shortness of breath. All participants provided written informed consent before enrolment. All participants were tested for HIV following Uganda’s HIV testing algorithm [18]; those diagnosed with HIV infection underwent CD4 cell count measurement. Demographic and clinical information was obtained by study personnel using standardized data forms.

### Collection and processing of samples

**Fig 1:**
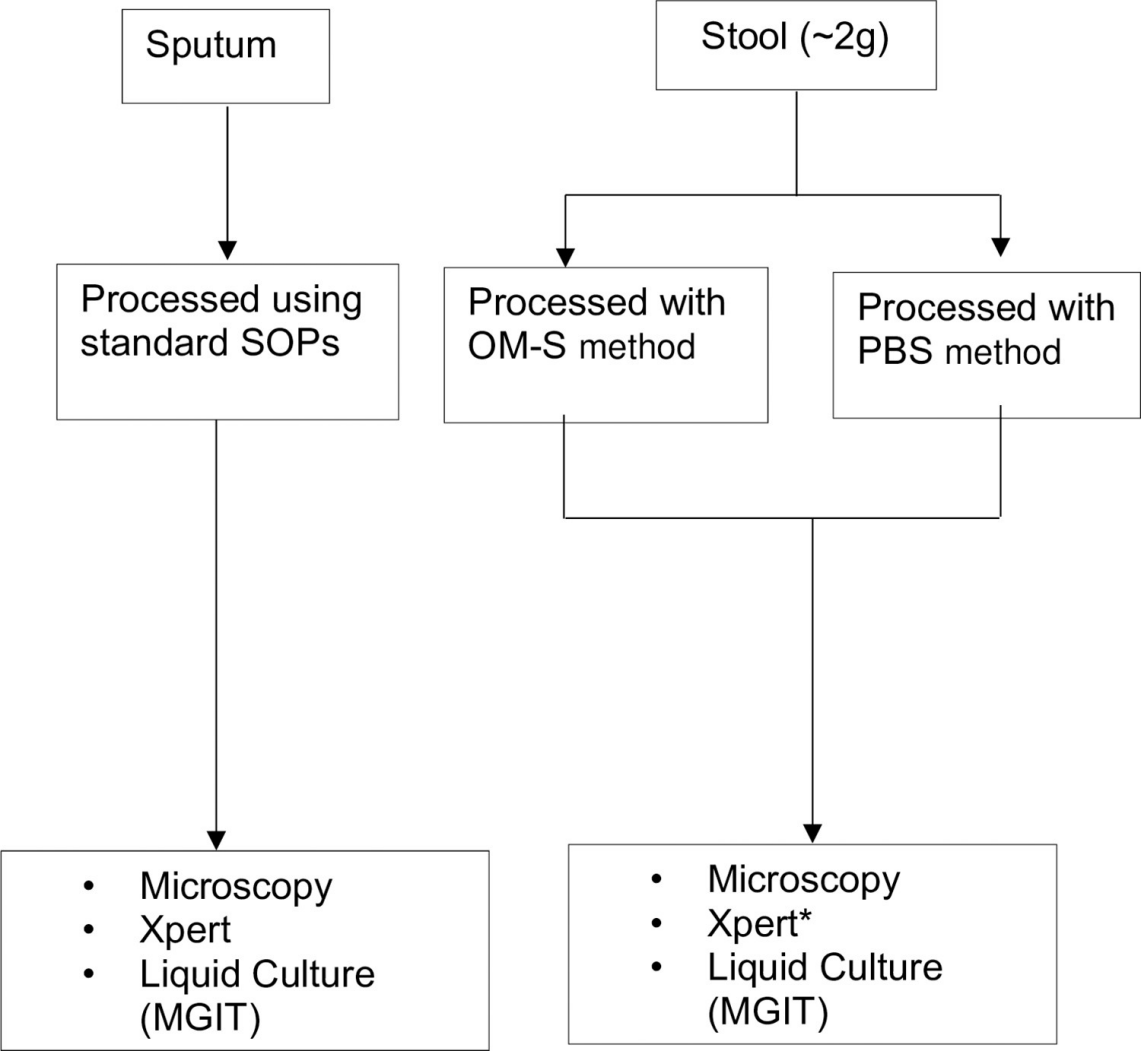
Flow of specimen collection, processing and testing. **Legend:** **MGIT**, Mycobacteria Growth Indicator Tube; **OM-S**, OMNIgene SPUTUM, **PBS**, Phosphate buffered saline, Xpert* Stool processed by PBS was not tested on Xpert.

Upon enrolment, participants were instructed to provide a stool (~2.0g) specimen on the spot and place it into a pre-labelled sterile container (Sarstedt Australia). A stool collection device was used to avoid mixing urine with stool. Half of the stool sample was processed using OM-S within 0.5 hours of collection and another processed using phosphate buffered saline (PBS) as a control **[Fig 1]**. Stool homogenate was incubated at ambient temperature for 15 minutes and washed twice by centrifuging at 3000g for 20 minutes. Sediment was re-suspended in 2-parts of PBS to obtain a final working sample, which was tested on microscopy, Xpert, and MGIT culture **[Fig 1]**. Stool homogenised in PBS was not tested on Xpert due to lack of funds.

Two expectorated or induced sputum samples were obtained one hour apart, decontaminated, and digested using the N-Acetyl-L-cysteine-NaOH method. Like stool, sputum samples were then centrifuged for 15 min at 3000g and sediment was later tested on microscopy, Xpert, and MGIT liquid culture [19] **[Fig 1]**.

### Laboratory tests

#### Microscopy

A smear of 1-2cm was prepared and air dried using 1ml of the homogenized stool or sputum sample. Smears were stained with 0.1% Auramine-O (Merck, Darmstadt, Germany), decolorized using 3% acid alcohol, and counterstained using 0.5% potassium permanganate before examination at 400X magnification using fluorescent microscopy. Results were recorded in the format outlined by the Clinical and Laboratory Standards Institute grading standards.

#### Xpert MTB/RIF Ultra

One volume (mL) of the OM-S stool or sputum sample was mixed with two volumes (mL) of the Xpert sample reagent, then loaded into cartridges and tested according to the manufacturer’s recommendations. Semi-quantitative results were recorded. Stool processed with OM-S was the only type tested on Xpert.

#### Liquid culture

Stool or sputum samples were decontaminated using NaOH/N-acetyl L-cysteine before incubation for a maximum of 42 days in MGIT culture media (BACTEC MGIT 960 System; Becton Dickinson, Franklin Lakes, NJ, USA). Culture contamination was assessed using blood agar (BACTEC 9120). Antigen MPT64 identification test was used to differentiate *M*. *tuberculosis* complex from nontuberculous mycobacteria. Time-to-culture-positive (TTP in days) was noted from the BACTEC MGIT 960 System. Results were reported according to International Union Against Tuberculosis and Lung Disease guidelines [20].

### Statistical analysis

We summarised demographic and clinical characteristics of the study population using frequencies and proportions for categorical variables while means and medians summarised continuous variables. We defined a bacteriologically confirmed PTB positive case as a case with a positive MGIT culture. For each index test, measures of diagnostic performance (sensitivity, specificity, and positive and negative predictive values) and associated 95% confidence intervals (CI) were calculated in STATA Version 12.0 (Stata Corp., College Station, TX) using MGIT sputum culture as the reference test. To control for misclassification bias due to culture contamination, we calculated sensitivity, specificity, positive and negative predictive values of stool MGIT using intention to diagnose sensitivity analysis. For each contaminated result, the final result was considered to be a false positive when the reference result was negative and a false negative when the reference result was positive [21].

### Ethical considerations

The study protocol was approved by Makerere University School of Medicine Research reference number (Ref) (2006–017), Uganda National Council of Science and Technology (Ref HS 259) Ethics Committees in Uganda and the University of California San Francisco Institutional Review Board in the USA (Ref 093055). Additional information regarding the ethical, cultural, and scientific considerations specific to inclusivity in global research is included in the (S2 File).

## Results

### Study population characteristics

**Fig 2:**
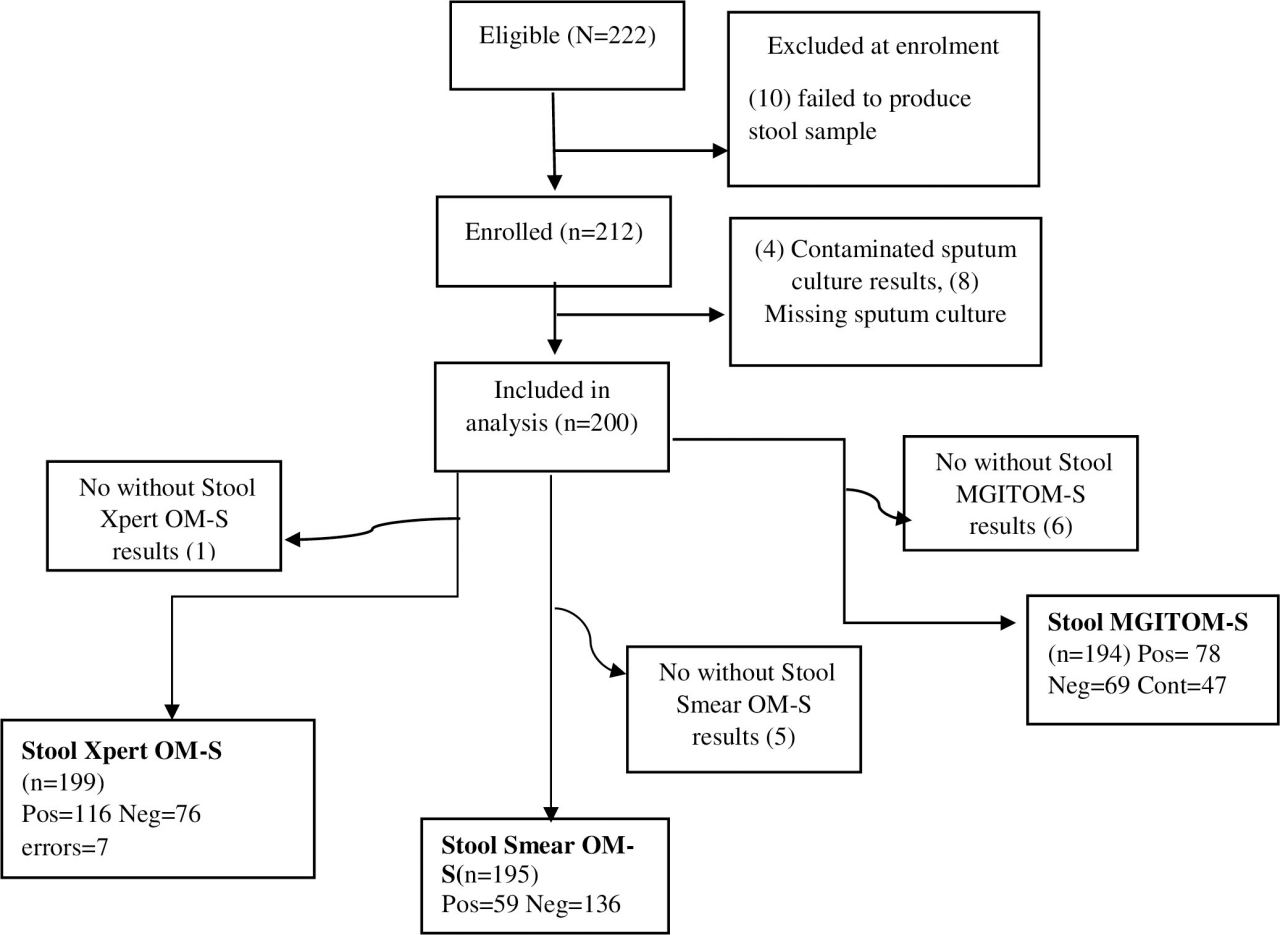
Study profile showing participants included and excluded from the study and total number and outcome of the OM-S stool samples per index test. **Legend:** **MGIT**, Mycobacteria Growth Indicator Tube; **OM-S**, OMNIgene SPUTUM, **Pos**, Positive; **Neg**, Negative; **Cont**, Contaminated.

Of the 222 eligible patients, 10 were excluded at enrolment and 12 at data analysis due to reasons stated in **[Fig 2]**. Among the 200 participants included in the analysis, median age was 33 (IQR 25–40) years **[Table 1]**, and the majority were male 120 (60%). Seventy-three (37%) were persons living with HIV, with median CD4 cell count of 120 cells/μl (IQR 43–297) and of these, 34 (47%) were already receiving antiretroviral therapy (ART) at enrolment. All participants had cough, including 85% who reported recurrent unexplained fever and 86% who noted weight loss as the most common complaints. One-hundred twenty-eight (64%) participants were bacteriologically confirmed to have pulmonary TB. Thirty-seven(29%) of the bacteriologically confirmed PTB positive participants were co-infected with HIV [**Table 1**].

**Table 1:** Demographic characteristics between bacteriologically confirmed TB patients and non-TB patients.

Characteristic	All participants	PTB^+^	PTB^–^
	N = 200	n = 128 (64%)	n = 72 (36%)
Age (yrs.), median IQR	33 (25–40)	31 (24–38)	35 (30–43)
Male gender, n (%)	120 (60)	85 (66)	35 (49)
Persons living with HIV, n (%)	73 (37)	37 (29)	36 (50)
Persons living with HIV on ART, n (%)^a^	34 (47)	16 (13)	18 (25)
CD4 (cells/μl), median (IQR)^b^	120 (43–297)	88 (40–193)	185 (66–401)
BMI, median (IQR)	19 (18–21)	19 (18–20)	21 (19–24)
Previous TB, n (%)	24 (12)	5 (4)	19 (26)
Smoking, n (%)	51 (26)	33 (26)	18 (25)
Alcohol use, n (%) **Clinical symptoms**	135 (68)	92 (72)	43 (60)
Cough >2weeks, n (%)	200 (100)	128 (100)	72 (100)
Weight loss>5%, n (%)	171 (86)	112 (88)	59 (82)
Fever, n (%)	170 (85)	115 (90)	55 (76)

**Abbreviations:** **ART** = Antiretroviral therapy; **BMI** = Body Mass Index; **CXR** = chest radiography; **HIV** = Human Immunodeficiency Virus; **IQR** = Interquartile Range; **PTB** = Pulmonary Tuberculosis.

**Legend:** ^b^CD4 were measured for HIV infected adults only; ^b^ART use was measured for HIV infected adults only.

### Performance of OM-S stool Xpert

Of the 199 OM-S stool samples tested on Xpert, 116 (58%) were positive for *Mtb*, 76 (38%) were Xpert negative and 7 (4%) were invalid errors **[Table 2]**. Additionally, 50 (43%) of the 116 stool Xpert positive results were graded as “low positive”. Only 1 result was identified as rifampicin resistant and 7 had indeterminate rifampicin resistant status. Four of the 7 samples that were indeterminate rifampicin resistant were also reported as “very low positive”, 1 low positive, and 2 were “trace positive”. OM-S stool Xpert detected an additional 35 and 55 *Mtb* positive cases compared to OM-S stool MGIT and OM-S stool microscopy, respectively.

**Table 2:** Results of stool and sputum samples per test.

Test	Total	Positive, n (%)	Negative, n (%)	Error/contaminated, n (%)
**Xpert MTB/RIF Ultra**				
OM-S stool	199	116 (58)	76 (38)	7 (4)
Sputum	177	110 (62)	67 (38)	0
**MGIT culture**
OM-S stool	194	78 (40)	69 (36)	47 (24)
PBS stool	169	45 (27)	21 (12)	103 (61)
Sputum	204	128 (64)	72 (36)	4 (2.0)*
**Microscopy**
OM-S stool	195	59 (30)	136 (70)	0
PBS stool	170	117 (69)	53 (31)	
Sputum	199	101 (51)	98 (49)	0

**Abbreviations:** MGIT = Mycobacteria Growth Indicator Tube (liquid culture) OM-S stool = stool homogenised in OMNIgene.SPUTUM, Xpert = Xpert MTB/RIF Ultra, PBS stool = stool homogenised in phosphate buffered saline.

**Legend:** * = Excluded from analysis.

Sensitivity (95%CI) and specificity (95%CI) of OM-S stool Xpert were 89% (83–94) and 91% (82–97), which were lower compared to sputum Xpert sensitivity and specificity of 95% (90–99) and 90% (86–96), respectively **[Table 3]**. Among the sputum smear negative PTB cases, sensitivity (95%CI) was highest for OM-S stool Xpert 65% (43–83) compared to stool MGIT at 39% (14–68) and stool smear at 12% (3–31) **[Table 4]**. Of the 47 contaminated OM-S stool MGIT samples, OM-S stool Xpert was able to identify accurately 21/25 (84%) samples as positive and 18/21 (86%) as negative when sputum MGIT was used as the reference. One sample was both culture-contaminated and had Xpert error.

**Table 3:** Performance of Xpert MTB/RIF Ultra, smear microscopy and MGIT and culture [Reference: MGIT-sputum].

	*Sensitivity*		Specificity		PPV		NPV	
	(95% CI)		(95% CI)		(95% CI)		(95% CI)	
**Xpert MTB/RIF Ultra**								
OM-S stool	89 (83–94)		91 (82–97)		95 (89–98)		83 (73–91)	
Sputum	95 (90–99)		90 (80–96)		94 (87–97)		92 (83–98)	
**Microscopy**								
OM-S stool	45 (36–54)		97 (90–100)		97 (88–100)		49 (41–58)	
PBS stool	50 (40–60)		99 (92–100)		98 (90–100)		56 (46–65)	
Sputum	80 (71–86)		97 (90–99)		98 (93–99)		73 (62–81)	
**MGIT culture**								
OM-S stool	60 (51–69)		64 (51–75)		74 (64–83)		48 (37–59)	
OMS stool*	75 (65–83)		92 (80–98)		95 (87–99)		64 (53–75)	
PBS stool	42 (32–52)		26 (16–38)		47 (36–54)		22 (13–33)	

**Abbreviations:** **MGIT** = Mycobacteria Growth Indicator Tube (liquid culture); **PPV** = positive predictive value; **NPV** = Negative predictive value; **95% CI** = 95% Confidence interval. **PBS stool =**, stool homogenised in phosphate buffered saline, **OM-S stool** = stool homogenised in OMNIgene.SPUTUM, **Xpert** = Xpert MTB/RIF Ultra.

**Legend:** *, Calculations made after excluding contaminated stool cultures.

**Table 4:** Performance of OM-S stool Xpert MTB/RIF Ultra, MGIT, and Smear microscopy among sputum smear negative samples.

Test	PTB-	PTB+	% Sensitivity	% Specificity
			(95% CI)	(95% CI)
**OM-S stool Xpert**				
Negative	63	8	65 (43–83)	91 (82–97)
Positive	6	15		
**OM-S stool MGIT**				
Negative	44	8	39 (14–68)	92 (80–98)
Positive	4	5		
**OM-S stool Microscopy**				
Negative Positive	67 2	22 3	12 (3–31)	97 (90–100)

**Abbreviations:** **MGIT** = Mycobacteria Growth Indicator Tube. **95% CI** = Confidence interval. **OM-S stool** = stool homogenised in OMNIgene SPUTUM; **Xpert**, Xpert = MTB/RIF Ultra. **PTB+** = Sputum culture positive; **PTB-** =, Sputum culture negative.

### Performance of OM-S stool MGIT

**Fig 3:**
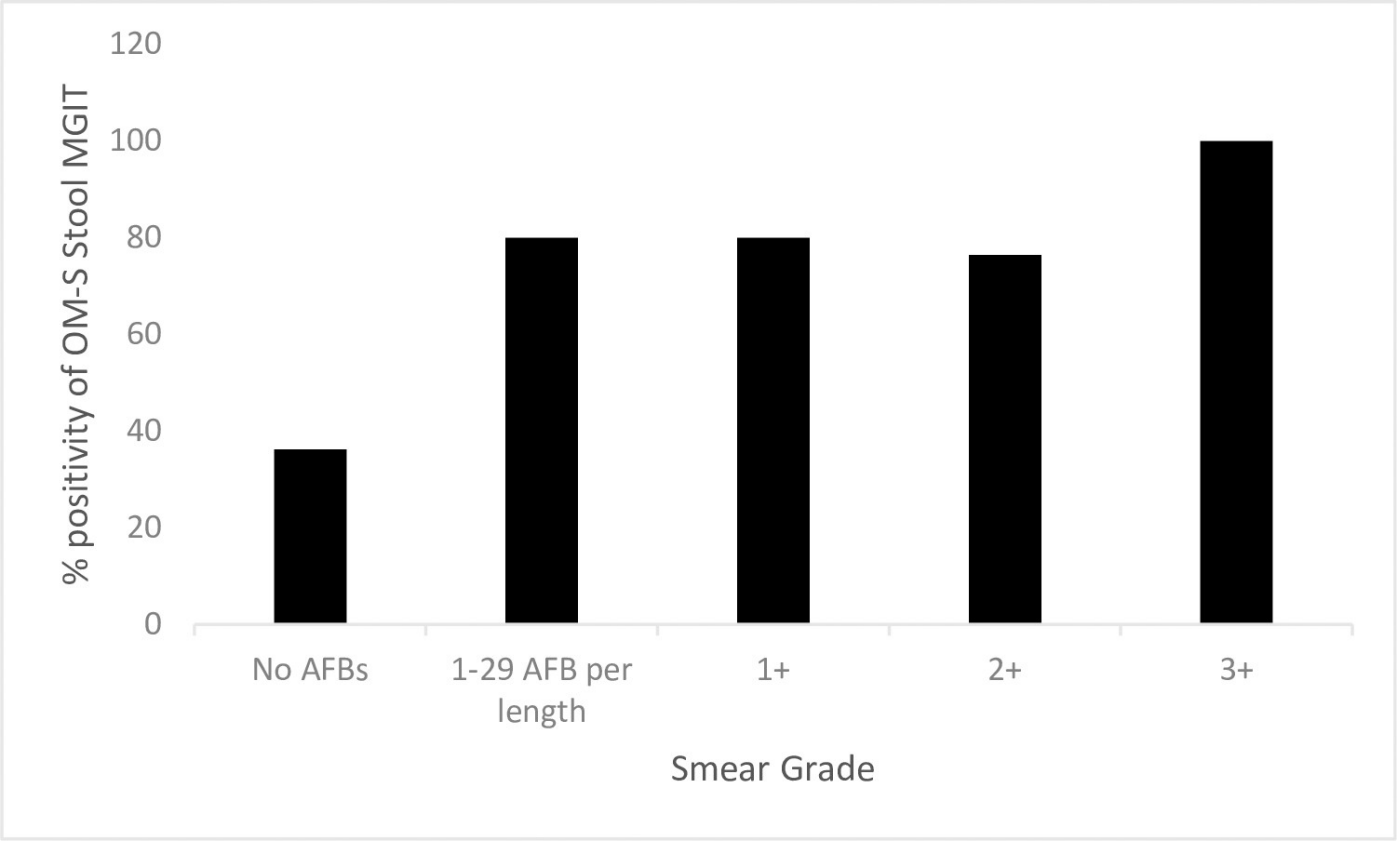
Percentage of positive stool MGIT OM-S culture per stool OM-S smear grade. Legend: No AFBS = 0 Acid Fast Bacilli (AFB)/30 fields, +1 = 30–299 AFB/30 fields, +2 = 10–100 AFB/field, +3 = >100AFB/Field.

We observed a 10-fold higher contamination rate for stool MGIT 47/194 (25%) compared to sputum MGIT 4/200 (2%) **[Table 2]**. Stool samples had a higher average MGIT time-to-positivity 12 days (IQR 8–18) compared to 7 days (IQR 4–11) for sputum MGIT culture. Among the non-contaminated stool and sputum samples, stool MGIT identified 74/99 (75%) PTB positive cases and 44/69 (64%) PTB negative cases, respectively. Sensitivity (95%CI) and specificity (95%CI) of stool MGIT after exclusion of contaminated results were 75% (65–83) and 92% (80–98), respectively **[Table 3]**. When contaminated samples were included in the denominator, sensitivity of stool MGIT was reduced from 75% to 60% and specificity from 92% to 64%, respectively, providing a conservative estimate of these tests **[Table 3]**. We noted that OM-S stool MGIT was more likely to be positive with an increasing level of stool smear grade. For example, the highest proportion of positive stool MGIT results were identified among participants with smear grades “3+” (4/4), followed by (13/17) among those with smear grade “2+” compared to (33/91) among those with smear negative **[Fig 3]**.

### Discordant OM-S stool MGIT results

We observed that of the 78 positive OM-S stool MGIT samples, only 4 samples tested negative by sputum MGIT; of these 3/4 corresponding sputum samples were negative on sputum Xpert, and 4/4 corresponding sputum samples were negative on sputum smear. We also noted that 19 OM-S stool MGIT results were negative, but were positive by OM-S stool Xpert. Of these, 17/19 (89%) had already been confirmed positive by sputum MGIT. Also noted were the 11 samples which were OM-S stool culture negative but stool smear positive, 9 of these were sputum MGIT positive.

### Performance of OM-S stool MGIT vs. PBS stool MGIT

OM-S processed stool samples had a higher positivity rate 78/194 (40%) compared to PBS processed stool 45/169 (25%) **[Table 2]**. A significant difference in contamination rates was observed among samples processed by PBS 103/169 (61%) compared those processed by OM-S processed stool 47/194 (24%) **[Table 2].** Both methods agreed on 15 (9%) negative, 36 (21%) positive and 31 (19%) contaminated samples. Fifty-six (29 negative and 27 positive) of the PBS stool MGIT contaminated samples, were accurately diagnosed by OM-S stool MGIT while PBS stool MGIT could only diagnose accurately 5 (3 negative and 2 positive) samples of contaminated OM-S stool MGIT samples when compared to sputum MGIT as reference. With inclusion of contaminated samples in the analysis a higher sensitivity 60% (95%CI 51–75) among OM-S processed samples compared to 42% (95%CI 32–52) was observed **[Table 3]**. A similar trend was observed with the specificity **[Table 3]**. Comparative analysis of sensitivity and specificity after exclusion of contaminated samples was not done due to high levels of contamination among the PBS processed samples and therefore high risk of misclassification bias.

### Diagnostic performance of OM-S stool Microscopy

Out of the 128 bacteriologically confirmed PTB cases, OM-S stool smear microscopy identified 57 (45%) accurately. Sensitivity of OM-S stool smear 45% (95%CI 36–54) was lower than 50 (95%CI 40–60) and 80% (95%CI 71–86) for PBS-stool and sputum smear, respectively **[Table 3]**. Specificity of OM-S stool smear at 97% (95%CI 90–100) was similar to that of sputum smear 97% (95%CI 90–99) however lower than that of PBS-stool 99% (95%CI 92–100) **[Table 3]**. Compared to stool Xpert and OM-S stool MGIT, stool smear showed the lowest levels of sensitivity **[Table 3]**. Both stool-smear and stool MGIT agreed on the positivity of 45 stool samples which were also confirmed positive by sputum MGIT. We observed that of the 47 contaminated stool MGIT samples, stool smear identified only 3/26 (12%) as positive and 21/21 (100%) as negative accurately basing on sputum MGIT as reference.

## Discussion

Our stool processing method was able to diagnose PTB with a higher stool MGIT culture sensitivity and a within range sensitivity of stool Xpert compared to previous methods. We also observed a lower stool culture contamination rate compared to our PBS processed stool control. Previous studies have shown stool samples as potential alternative to respiratory specimens for *Mtb* diagnosis among patients who cannot easily provide sputum [8,9,22,23]. If successfully achieved, clinical use of stool for PTB diagnosis would eventually enhance achievement of the END TB strategy target of 80% reduction in the TB incidence rate by 2030. Use of stool for TB diagnosis however, has faced limitations of low *Mtb* yields, molecular diagnostic test errors and high rates of culture contamination [23]. Our stool processing method aimed at reducing Xpert MTB/RIF errors and culture contamination but with minimal adverse effect on viable *Mtb* yield.

Our findings demonstrated that when using our processing method, Xpert MTB/RIF was still the most sensitive standard diagnostic TB test on stool samples. Our sensitivity (89%) was higher than some of previous studies [8,10,13–15,24,25] and consistent with findings of [9,22]. Use of paediatric patients as study population in studies [10,13,14] and differences in variety of stool processing approaches for studies may have led to differences in sensitivity. A study by Hilleman, et. al. [6] reported 100% sensitivity of stool Xpert MTB/RIF; however, they only used a sample size of 23 patients and therefore a possibility of random bias cannot be ruled out. In our study, stool Xpert identified 19 additional TB positive stool samples compared to stool MGIT, and 62 more compared to stool microscopy. This difference may be due to the lower numbers of viable culturable or stainable *Mtb* bacteria caused by the harsh acidic conditions of gastrointestinal tract system in addition to stool sample processing methods compared to *Mtb* DNA identified by Xpert MTB/RIF whether dead or viable. These findings further emphasize Xpert MTB/RIF as currently the most suitable test for diagnosis of stool *Mtb* hence the recent WHO recommendation to use stool Xpert for initial diagnosis of TB among children [26]. Our stool Xpert Sensitivity was higher than that of sputum microscopy (80%) with stool Xpert able to identify 15/23 PTB positive samples accurately among sputum smear negative samples. This may be an indication that testing stool processed by our method on Xpert may have an important role for diagnosis of sputum smear negative *Mtb*. Due to the low numbers of sputum smear negative PTB patients in our study, we cannot rule out the possibility of random bias in our findings. We therefore recommend evaluation studies of our stool processing method on Xpert among a bigger sample size of sputum smear negative PTB patients.

Stool Xpert error results are due to inhibition of DNA Taq polymerase enzyme by faecal inhibitors leading to failure of Xpert PCR amplification [27,28]. Prior studies [6,8] reported 13% invalid stool Xpert results and 10% invalid stool RD9-based PCR results, respectively compared to our Xpert error rates (4%). Other studies [22,29,30] had error rates of (3.2%, 2.6%, and 2%, respectively) consistent with our findings an indication our method may be reliable in removing of faecal PCR inhibitors.

Sputum Xpert was still more sensitive (94%) compared to our stool Xpert (89%). This difference may be explained by the fact that detecting *Mtb* DNA in stool is dependent on patients swallowing of enough volume of *Mtb* infected sputum into the GI tract compared to using of sputum with high bacterial load directly from the lungs for *Mtb* diagnosis. Our hypothesis is supported by our findings which show that the higher the sputum microscopy smear grade the higher the likelihood for a positive stool Xpert and MGIT. Because we collected on spot stool samples on the same day of collecting sputum samples, we think some of the PTB positive patients enrolled in the study may not have swallowed enough sputum by the time we accessed the stool sample.

A lower sensitivity was observed for our OM-S processed (46%) stool microscopy as compared to other stool index tests. This was consistent with sensitivity of sputum microscopy compared with other sputum index test as reported by others [17,31]. Our stool microscopy sensitivity was lower than that of with Rahman, et. al. [22] (53%) although was still higher sensitivity compared to Amel El Khéchine, et. al. (35%) [7] and (12%) by Abaye, et. al.

The ease with which microscopy can be conducted makes the method the most available *Mtb* test [32] and despite the relatively low sensitivity microscopy may still play a role in diagnosis of *Mtb* in hard to reach areas where molecular assays like Xpert may not be available.

Culture is the *Mtb* gold standard test and the most sensitive in the diagnosis of pauci-bacillary TB [33,34]. Understanding *Mtb* culture performance on stool therefore would be important for clinical management. High level stool culture contamination rates [11,23] caused by several non-TB microflora which grow more rapidly in culture than *M*. *tuberculosis* bacilli is a major challenge in utilization of stool culture for *Mtb* diagnosis. Our stool processing method had lower culture contamination rates compared to our control and studies by [6,23]. We believe decontamination characteristics of OM-S as reported by [17] were responsible for this difference in contamination rates. Study findings of [11,12,22] reported similar or lower contamination rates to our findings however these had a limitation of small sample size and lower sensitivity (35.3%, 44.0%, and 31.9%, respectively) compared to our findings. The lower contamination rate relative to lower sensitivity may indicate failure to balance between stool decontamination and maintenance of *Mtb* viability during stool processing. Using our findings, we believe our OM-S based processing protocol may have potential to optimize and balance these two characteristics despite a higher time to culture positivity than sputum.

Our OM-S processed stool culture sensitivity (76%) after exclusion and (60%) with inclusion of contaminated samples in analysis respectively to our knowledge is this highest *Mtb* sensitivity for any stool culture study among adults with PTB compared to the next highest sensitivity of 54% by Amel El Khéchine, et. al. [7] who used 3 volumes of 1% chlorhexidine digluconate (Sigma) for stool decontamination. Our results highlight the potential of utilization OM-S reagent for stool processing to improve yield of viable *Mtb* in when using stool culture for diagnosis despite the harsh acidic environment of stool.

Stool Xpert however was able to accurately diagnose 85% the culture contaminated samples when we used sputum MGIT as a reference. Stool MGIT on the other hand could only diagnose accurately 57% (4/7) of the stool Xpert errors stool. The relationship may be an indication that with our stool processing method clinicians may benefit in using both methods with stool culture being opted in high-risk patients who have had Xpert errors or a negative stool Xpert result in addition to patients who critically need drug susceptibility testing results to rule out or treat Multi drug resistant TB. We did not find any studies done to assess benefit of this conditional utilization of both methods. This study did not have the power to investigate this objective and thus we recommend future studies to evaluate the benefit and feasibility of using both stool Xpert and culture among the categories of patients mentioned above.

One of the limitations of our study is that we did not include paediatric or severely sick adult patients both of whom would best benefit from this study. Before applying our stool processing method to paediatric or severely sick patients we needed to validate if our method can detect *M*. *tuberculosis* from stool sample of sputum producing bacteriologically positive patients when tested on standard of care *M*. *tuberculosis* tests. Our findings provide insightful information of a potential TB diagnostic stool processing method for future studies in paediatric population and other patients with difficulty to expectorate. During analysis, we did not adjust for the possibility of extra-pulmonary TB such as GI or lymph node TB to which some of our results may be attributed especially in *Mtb* positive stool samples which did not have any positive corresponding sputum tests. A single Rifampicin resistant sample we identified was not tested for DST and thus could not understand the utility of our method to diagnose RIF resistance when using stool Xpert. Due to limited study funds, we had logistical challenges to procure enough Xpert cartridges to test stool homogenized in PBS on Xpert. We thus did not have a comparator for our stool processing method tested on Xpert. This limited our ability to confidently attribute stool Xpert outcomes on our processing method. We however anticipate that publication of our findings will demonstrate the potential and importance of this additional test during similar future studies.

## Conclusions

Our stool processing method demonstrated high potential to improve *Mtb* diagnosis in populations with difficulty in sputum production using both stool molecular and culture tests. With WHO now recommending use of stool Xpert for diagnosis of PTB in children less than 10 years old, focus should now be on how best to process stool for culture for better *Mtb* yields with less contamination. We think our method has attempted to balance both these characteristics although the culture contamination rates are still very high compared to sputum culture and thus, we cannot suggest replacing our stool culture with sputum culture in the diagnosis of PTB. We however believe our method still has a role in diagnosis of high-risk patients such as paediatric patients at risk of MDR-TB and HIV+ patients who cannot benefit from stool Xpert. We recommend future studies of this protocol to be done directly among paediatric cases, drug resistant and severely sick patients who cannot produce sputum.

## Supporting information

S1 File Dataset for the study. (XLSX)

S2 File Checklist. (DOCX)
